# Prepectoral breast reconstruction with complete anterior implant coverage using a single, large, square-shaped acellular dermal matrix

**DOI:** 10.1186/s12893-022-01683-z

**Published:** 2022-06-20

**Authors:** Hyun Ki Hong, Yun Hyun Kim, Joon seok Lee, Jeeyeon Lee, Ho Yong Park, Jung Dug Yang

**Affiliations:** 1 Department of Plastic and Reconstructive Surgery, School of Medicine, Kyungpook National University, 130 Dongdeokro, Jung-gu, Daegu, 41944 Korea; 2 Department of Surgery, School of Medicine, Kyungpook National University, 130 Dongdeokro, Jung-gu, Daegu, 41944 Korea

**Keywords:** Prepectoral, Anterior coverage, Implant insertion, Comparative cohort study

## Abstract

**Background:**

Several studies have discussed various methods of prepectoral direct-to-implant (DTI) breast reconstruction using an acellular dermal matrix (ADM) prosthesis to achieve full coverage. However, methods for anterior coverage have rarely been reported. In this study, prepectoral DTI breast reconstruction with complete anterior implant coverage was performed using a square piece of ADM. This study aimed to introduce our prepectoral DTI technique and determine its functional and cosmetic outcomes as well as compare them with those of existing subpectoral DTI techniques.

**Methods:**

This prospective comparative study focused on 29 patients (35 breasts) and 34 patients (35 breasts) who underwent breast reconstruction via subpectoral implant insertion (control group) and anterior coverage prepectoral implant insertion (anterior coverage group), respectively. Postoperative complications were noted, and breast symmetry was evaluated using the Vectra H2 three-dimensional scanner. The modified Kyungpook National University Hospital Breast-Q (KNUH Breast-Q) scale was used to assess the patient’s subjective satisfaction with the reconstruction and postoperative quality of life.

**Results:**

No remarkable differences in terms of complications (seroma, skin necrosis, nipple–areola complex necrosis, hematoma, capsular contracture, and infection) were noted in both groups. Compared with controls, considerably better results were observed among those in the anterior coverage group in terms of the mean drain removal period. Furthermore, those in the anterior coverage group showed greater symmetry on three-dimensional scans than the controls; however, this was not statistically significant. Subjective satisfaction and postoperative quality of life measured using the KNUH Breast-Q scale were not significantly different between both groups.

**Conclusions:**

Considering its stability, faster recovery time, and cosmetic benefit, prepectoral breast reconstruction with anterior implant coverage using a single, large ADM is a good choice to perform breast reconstruction with implant insertion in patients who have undergone mastectomy.

*Level of evidence*: II.

**Supplementary Information:**

The online version contains supplementary material available at 10.1186/s12893-022-01683-z.

## Introduction

Breast cancer is the most common cancer occurring in women, and its prevalence increases each year [1]. With the emergence of Breast CAncer gene testing, prophylactic mastectomy is also becoming more common [2]. Therefore, patients who wish to resolve the defects caused by mastectomy and restore their cosmetic and functional aspects to normal can undergo breast reconstruction [2].

Generally, breast reconstruction can be performed using autologous tissue and prostheses. Direct-to-implant (DTI) breast reconstruction is a prosthesis technique that is widely used, and it is more common than autologous breast reconstruction. Thus, obtaining a deeper understanding of this technique is imperative [3]. Among DTI breast reconstructions, the subpectoral dual-plane technique was used to a large extent in the past; however, in recent years, prepectoral DTI breast reconstruction has become more popular [2, 4]. Various methods and materials have been involved in the development of prepectoral DTI breast reconstruction [4–7], and a material called acellular dermal matrix (ADM) has contributed massively to its development [2, 4]. Recently, various methods of prepectoral DTI breast reconstruction have been used to achieve implant coverage using ADM. These methods have the ability to preserve the pectoralis major muscle [8, 9], thereby reducing pain, animation deformity, and recovery time [10]. Furthermore, compared with subpectoral DTI breast reconstruction, prepectoral DTI breast reconstruction is reported to have good cosmetic outcomes and a low occurrence of capsular contractures because of the ease of adjusting the position of the inframammary fold (IMF) [11–14].

Although prepectoral DTI breast reconstruction uses various methods and has numerous advantages in several aspects, no established consensus exists on the optimal choice of technique. Several studies have been conducted on the methods of prepectoral DTI breast reconstruction using ADM. Various techniques using a large piece of ADM to achieve full prosthesis coverage have been reported [15, 16]. We have previously reported about this method by applying two ADMs crosswise (the two double-crossed ADM method) [9].

In addition to full prosthesis coverage, another method involves covering the prosthesis anteriorly (anterior coverage); however, few studies have been reported in this regard. As a method to achieve anterior coverage, our team performed prepectoral DTI breast reconstruction through complete anterior coverage of the implant using a single, large, square-shaped piece of ADM. The objective of this study was to introduce our prepectoral DTI technique and compare its functional and cosmetic outcomes with those of the existing classic subpectoral DTI technique.

## Methods

### Patients and data

The institutional review board of Kyungpook National University Hospital (Kyungpook National University Medical Center No. 2019-03-017) approved the study protocol. We performed a non-randomized prospective comparative study to identify patients who had undergone single-stage prepectoral breast reconstruction with complete anterior implant coverage using a single, large, and square-shaped ADM (anterior coverage group) and those who underwent surgery using the subpectoral DTI technique (control group), with all surgeries performed by a single, senior surgeon. If the skin flap was thin and concern existed about skin necrosis after the patient underwent mastectomy conducted by a breast surgeon, surgery was performed using the subpectoral plane. All other surgeries were performed using the prepectoral plane. Between January 2019 and December 2020, the anterior coverage method was used in 35 breasts among 34 consecutive patients, whereas the subpectoral method was used in 35 breasts among 29 consecutive patients.

We collected the following patient data: date of surgery, age at the time of surgery, body mass index, cancer staging, and whether radiation therapy had been performed (and timing, if radiation therapy performed). Relevant complications and their specific types were also investigated.

### DTI breast reconstruction operative technique

#### Classic subpectoral DTI breast reconstruction

The most suitable prosthesis was chosen based on the width and volume of the breasts and by measuring the weight of the excised mass immediately after mastectomy. To create a pocket for prosthesis insertion, pectoralis major muscle dissection was carefully performed by minimizing bleeding and damage to the surrounding tissue. For the portion that had insufficient coverage with an inferior part of muscle, the pocket for the prosthesis was completed using the ADM (Bellacell HD™, HANSCARE, Seoul, South Korea).

#### Prepectoral DTI breast reconstruction (anterior coverage)

**Fig. 1:**
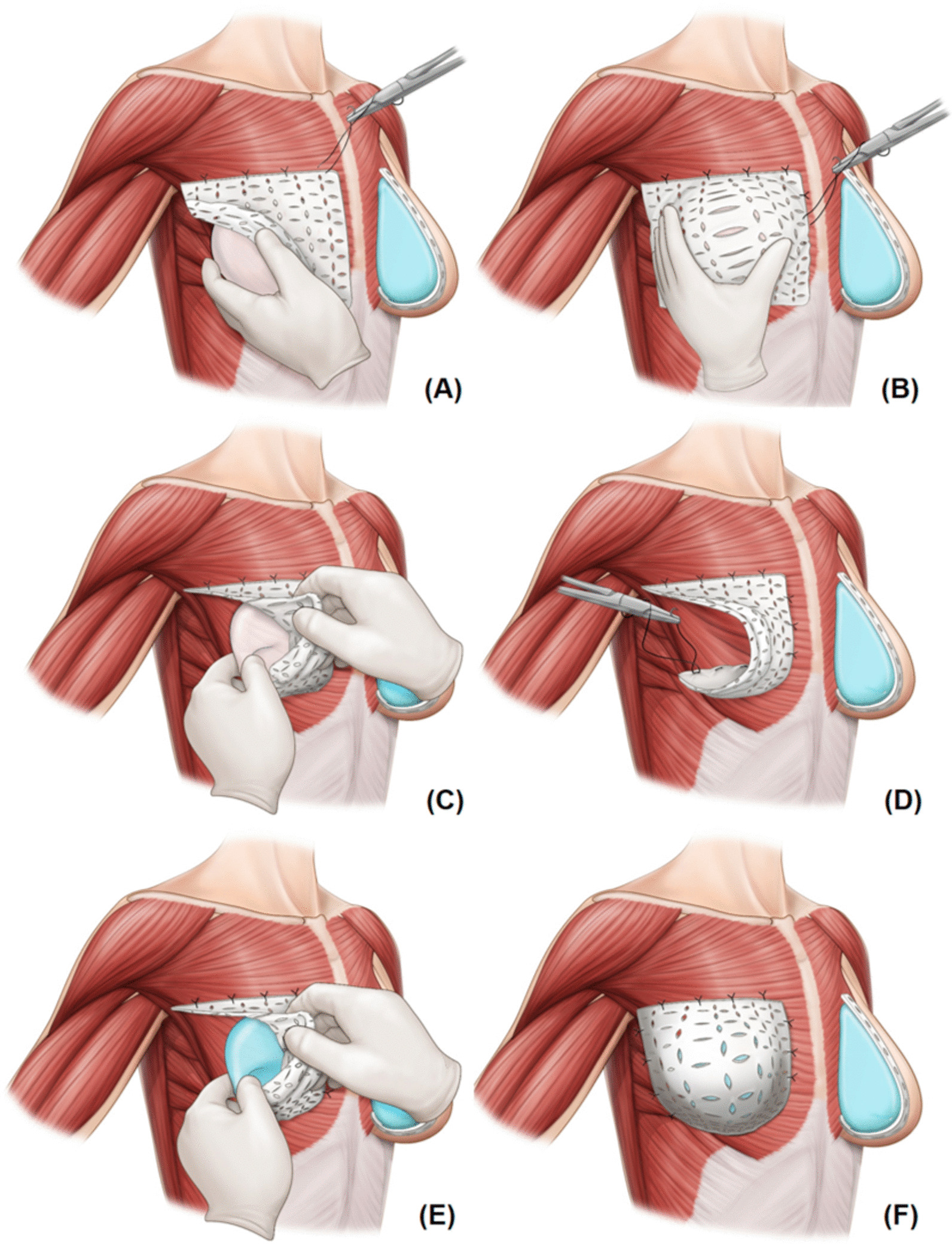
Surgical technique used for anterior coverage. **a** After inserting an appropriate implant sizer, the acellular dermal matrix (ADM) was sutured from the superior margin. **b** After retaining the implant sizer, the medial border of the ADM was fixed to the pectoralis major. **c** The ADM was fixed by folding the ADM downward into the implant sizer to form a natural inframammary fold, after which the implant sizer was removed. **d** The inferior margin of the ADM was sutured, leaving an opening on the lateral aspect to place the implant. **e** Insertion of the implant. **f** After implant placement, the ADM provides total anterior coverage of the prosthesis

This anterior coverage technique uses a 16- × 16-cm^2^ sheet of ADM (Bellacell HD, HANSCARE), which is modified according to the size of the breast pocket and implant. The ADM was prepared on a separate sterile table followed by rehydration in iodine and normal saline according to the manufacturer’s instructions. Prepectoral pocket formation was initiated by insetting the ADM over the pectoralis muscle with interrupted sutures using 2/0 Vicryl^®^ (Ethicon, Raritan, NJ, USA). To choose the appropriate implant size for breast reconstruction, an implant sizer was used. Insertion of the appropriate implant sizer was followed by fixing the superior border of the ADM to the pectoralis major. On leaving the implant sizer in, the medial border of the ADM was fixed to the pectoralis major. The location of the inferior aspect of ADM fixation was chosen to form a natural IMF by folding the ADM downward on the implant sizer. The ADM was sutured on the inferior aspect, leaving an opening on the lateral aspect for placing the implant. The ADM in the inferior aspect was curved along the IMF perimeter to re-create the natural curvilinear contour of the breast with the patient repositioned in a 45° upright position for a more realistic assessment of gravitational forces on the implant and soft-tissue envelope. After inserting the prosthesis of an appropriate size selected using the implant sizer, the lateral aspect was sutured according to the breast contour. After implant placement, the ADM provided complete anterior coverage of the prosthesis (Fig. 1). The nipple-sparing mastectomy flaps were then tailored as necessary and closed in layers. Additional file 1: Video 1 shows the surgical technique used for prepectoral breast reconstruction with complete anterior coverage of the implant using a single, large, square-shaped ADM.

### Clinical outcome

**Fig. 2:**
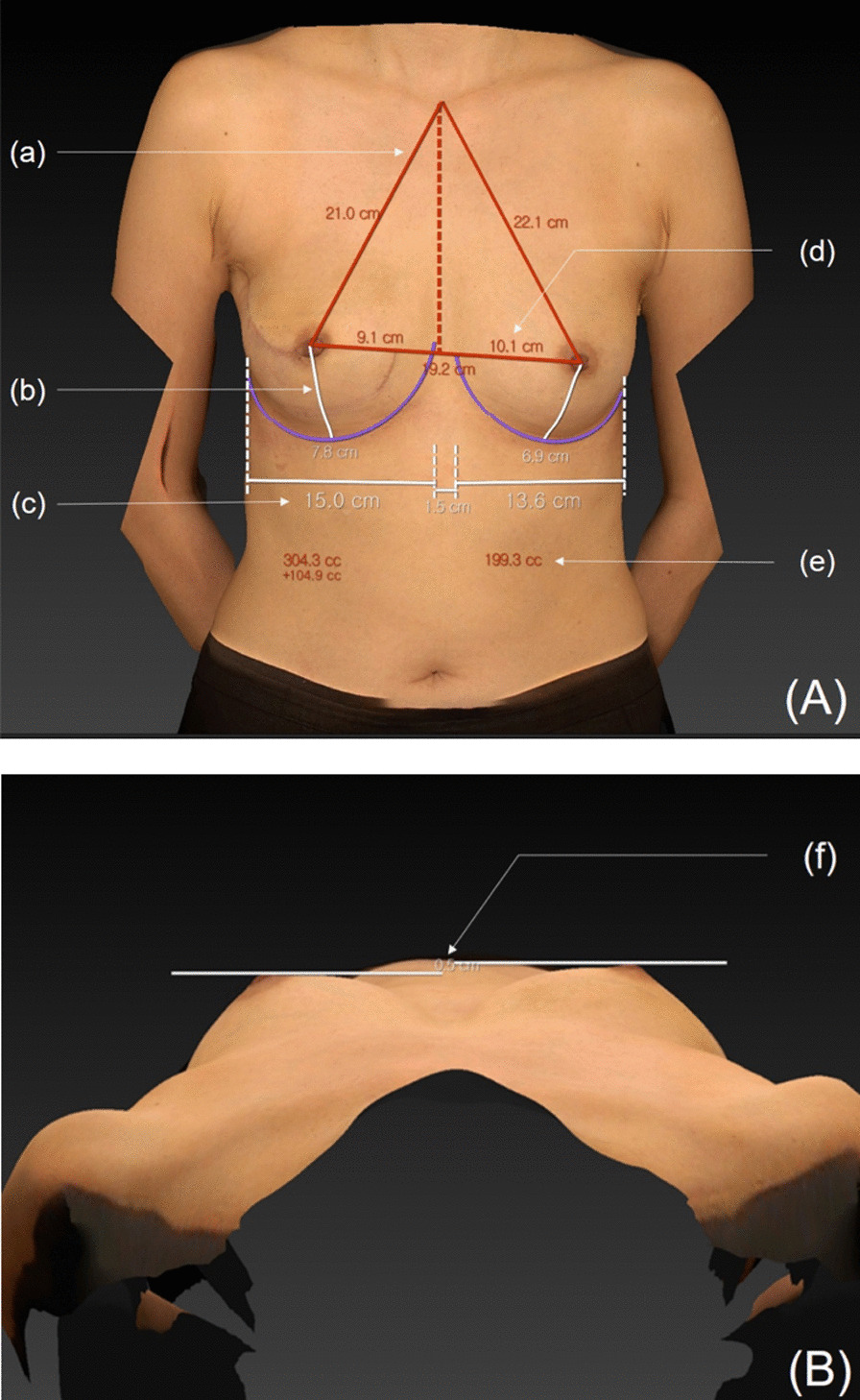
Measurement of symmetry parameters using the Vectra H2 3D scanner. **A** Anterior–posterior view. **a** Sternal notch to nipple (SN–N) distance. **b** Inframammary fold line to nipple (IMF–N) difference. **c** Breast width. **d** Nipple to midline (N–M) distance. **e** Breast volume. **B** Craniocaudal view. **f** Breast projection difference

Among the patients who underwent subpectoral implant insertion and those who underwent prepectoral implant insertion with complete anterior coverage, the number and percentage of individuals who experienced seroma, linear skin necrosis, hematoma, capsular contracture, infection, and rippling were noted. Complications were categorized as major (requiring rehospitalization or surgical treatment) and minor (can be treated through outpatient treatment). Among minor complications, seroma was considered when fluid was considered enough to require aspiration. The patients’ subjective satisfaction with respect to reconstruction and quality of life (QoL) after surgery was evaluated using the modified Kyungpook National University Hospital Breast-Q (KNUH Breast-Q) scale. Breast symmetry was measured postoperatively using the Vectra H2 three-dimensional (3D) scanner (Canfield Scientific, Inc., Parsippany-Troy Hills, NJ, USA). Using this scanner to compare the level of symmetry in both groups, we measured the difference between the shortest distance of each sternal notch to the nipple (SN–N) and that of each IMF to the nipple (IMF–N), the difference in breast width between breasts, the difference in the nipple to midline (N–M) distance between breasts, the difference in breast projection between breasts, and the volume difference between breasts (Fig. 2). In addition, the cosmetic outcome was confirmed through gross photos captured during the postoperative follow-up period. All follow-up was performed by a single, senior surgeon.

### Statistical analysis

A p-value of < 0.05 indicated statistical significance. Statistical analysis was performed using IBM SPSS version 23.0 (IBM Corp., Armonk, NY, USA). A Chi-squared test or Fisher’s exact test was used for categorical variables, whereas a two-sample *t* test was used for continuous variables.

## Results

A total of 63 patients with breast cancer underwent nipple-sparing mastectomy between January 2019 and December 2020. Our prospective comparative study included 29 patients (35 breasts) and 34 patients (35 breasts) who underwent breast reconstruction through subpectoral implant insertion and prepectoral implant insertion, respectively, using the complete anterior coverage method. Table 1 presents the patient demographic information.

**Table 1:** Patient characteristics^a^

	Subpectoral (n = 29)	Prepectoral (n = 34)	p value*
No. of breasts	35	35	
Age, years, mean ± SD (range)	43.8286 ± 7.9428	47.2353 ± 10.6716	0.0693
≥ 0, N (%)	2 (6.9%)	0 (0%)	
≥ 30, N (%)	4 (13.8%)	2 (5.9%)	
≥ 40, N (%)	21 (72.4%)	20 (58.8%)	
≥ 50, N (%)	2 (6.9%)	10 (29.4%)	
≥ 60, N (%)	0 (0%)	2 (5.9%)	
> 70, N (%)	0 (0%)	0 (0%)	
BMI, kg/m^2^, mean ± SD (range)	22.2214 ± 2.6532 (10.1751)	22.0161 ± 2.3392 (9.8872)	0.3670
Excised mass weight g, mean ± SD (range)	318.8571 ± 122.2146 (292.1565)	286.4706 ± 119.7632 (303.7712)	0.0752
Silicone implant volume, cc, mean ± SD (range)	346.7143 ± 169.202 (301.7834)	284.4118 ± 157.1419 (310.9185)	0.0588
Cancer staging, n (%)			0.3494
0	9 (31.2%)	7 (20.5%)	
I	15 (51.7%)	17 (50%)	
II	3 (10.3%)	9 (26.6%)	
III	2 (6.8%)	1 (2.9%)	
IV	0 (0%)	0 (0%)	
Chemotherapy, n (%)			0.1457
None	22 (75.8%)	12 (35.3%)	
Neoadjuvant only	0 (0%)	0 (0%)	
Neoadjuvant and adjuvant	0 (0%)	0 (0%)	
Adjuvant only	7 (24.2%)	22 (64.7%)	
Adjuvant hormone therapy	18 (62.5%)	22 (64.7%)	
Radiotherapy, n (%)			0.7598
None	25 (86.2%)	31 (91.20%)	
Neoadjuvant	0 (0%)	0 (0%)	
Adjuvant	4 (13.8%)	3 (8.8%)	

^a^*BMI* body mass index

The complication rate was compared between the two groups, and the respective results of the control and anterior coverage groups are detailed below. The average follow-up period (mean ± standard deviation (SD)) was 19.9 ± 2.2274 months in the control group and 16.3 ± 8.8851 months in the anterior coverage group. No major complications were observed in either of the groups. The following minor complications were observed in the control and anterior coverage groups, respectively: seroma (five patients [14.29%] and three patients [8.57%]), linear skin necrosis (five patients [14.29%] and three patients [8.57%]), hematoma (zero patients [0%] in both groups), capsular contracture (one patient [2.86%] and zero patients [0%]), infection (one patient [2.86%] and zero patients [0%]), and rippling (zero patients [0%] and two patients [5.71%]). No statistically significant differences in terms of complications were noted in both groups.

The mean period of drain removal (mean ± SD) was 11.1143 ± 2.8468 and 8.5588 ± 2.5008 days (p < 0.001) in the control and anterior coverage groups, respectively, revealing a statistically significant difference (Table 2).

**Table 2:** Complication rate

	Subpectoral (n = 29)	Prepectoral (n = 34)	p value*
No. of breasts	35	35	
Mean follow-up period, months, mean ± SD (range)	19.8771 ± 5.9963 (4.8812)	16.2931 ± 8.8851 (3.9975)	0.2750
Major complication, n (%)			
Reoperation	0 (0%)	0 (0%)	
Minor complication, n (%)			
Seroma	5 (14.29%)	3 (8.57%)	0.2423
Linear skin necrosis	5 (14.29%)	3 (8.57%)	0.1551
Hematoma	0 (0%)	0 (0%)	
Capsular contracture	1 (4.76%)	0 (0%)	0.1622
Infection	1 (4.76%)	0 (0%)	0.1622
Rippling	0 (0%)	2 (5.71%)	0.5188
No complication	22 (62.86%)	25 (71.43%)	0.1741
Mean drain removal period, days, mean ± SD (range)	11.1143 ± 2.8468 (4.4471)	8.5588 ± 2.5008 (3.8124)	0.0275*

Number and percentage of patients who experienced complications were recorded. Complications are mainly categorized as either major (i.e., requiring rehospitalization or surgical treatment) or minor (i.e., can be treated through outpatient treatment) complication

^*^p < 0.05

We evaluated the subjective satisfaction of patients regarding reconstruction and QoL after the surgery using the KNUH Breast-Q scale. No statistical significance was observed in any of the items (Table 3).

**Table 3:** Patient satisfaction and quality of life evaluated using the modified KNUH University Hospital Breast-Q at 12 months postoperatively

	Very satisfied	Subpectoral (n = 29)	Prepectoral (n = 34)	p value*
1. Overall, are you satisfied with your breast reconstruction?	5	4.3	4.3	0.125
2. Are you satisfied with breast symmetry achieved after reconstruction?	5	4.0	4.1	0.482
3. Are you satisfied with the size of your breast after reconstruction?	5	4.2	4.3	0.247
4. Are you satisfied with the shape of your breast after reconstruction?	5	3.9	4.0	0.542
5. Are you satisfied with how your breasts feel after reconstruction?	5	4.2	4.0	0.127
6. Are you satisfied with the level of pain you had to endure after reconstruction?	5	4.3	4.2	0.424
7. Are you satisfied with the scar resulted after breast reconstruction?	5	3.7	3.9	0.542
8. Have you experienced a loss of confidence or self-esteem after breast reconstruction?	5	4.1	4.3	0.984
9. Are you satisfied with your sexual attractiveness after breast reconstruction?	5	4.1	4.2	0.654
Total	55	44.8	45.2	0.214

^*^p < 0.05

We measured postoperative breast symmetry using the Vectra H2 3D scanner. In the control and anterior coverage groups, the differences between the shortest SN–N distance (mean ± SD) were 0.7783 ± 0.6510 and 0.5882 ± 0.5944 cm (p = 0.1048), between the shortest IMF–N distance were 0.8 ± 0.4802 and 0.7176 ± 1.1269 cm (p = 0.3482), between breast widths were 1.0229 ± 0.7670 and 0.7471 ± 0.7886 cm (p = 0.0728), between the shortest N–M distance were 0.9971 ± 0.5090 and 0.9441 ± 0.7468 cm (p = 0.3662), between breast projections were 0.6143 ± 0.3805 and 0.4853 ± 0.3322 cm (p = 0.0690), and between breast volumes were 38.3814 ± 28.2869 and 36.9559 ± 27.6897 cm (p = 0.4166), respectively. Although the overall difference was lower in the anterior coverage group than in the control group, no statistical significance was observed in either of the groups regarding all items (Table 4).

**Table 4:** Symmetry measurement using 3D scanner

	Subpectoral (n = 29)	Prepectoral (n = 34)	p value*
No. of breasts	35	35	
SN–N distance difference, cm, mean ± SD (range)	0.7783 ± 0.6510 (1.4022)	0.5882 ± 0.5944 (1.5024)	0.1048
IMF–N distance difference, cm, mean ± SD (range)	0.8 ± 0.4802 (0.8055)	0.7176 ± 1.1269 (0.6411)	0.3482
Breast width difference, cm, mean ± SD (range)	1.0229 ± 0.7670 (1.8412)	0.7471 ± 0.7886 (2.4176)	0.0728
N–M distance difference, cm, mean ± SD (range)	0.9971 ± 0.5090 (1.9054)	0.9441 ± 0.7468 (1.3071)	0.3662
Breast projection difference, cm, mean ± SD (range)	0.6143 ± 0.3805 (1.5412)	0.4853 ± 0.3322 (1.2411)	0.0690
Breast volume difference, cc, mean ± SD (range)	38.3814 ± 28.2869 (40.3074)	36.9559 ± 27.6897 (37.8123)	0.4166

The difference between the shortest distance of each sternal notch to the nipple (SN–N), that of each IMF to the nipple (IMF–N), the difference in projection between both breasts, and the volume difference between both breasts were measured using the Vectra H2 (Canfield Scientific, Inc.) 3D scanner to compare the level of symmetry between both groups^a^

^a^SN–N, sternal notch to nipple; IMF–N, inframammary fold line to nipple; N–M, nipple to midline

^*^p < 0.05

## Discussion

Among the methods used for breast reconstruction using a prosthesis, prepectoral breast reconstruction is known for its stability and cosmetic outcomes. Sigalove et al. reported that prepectoral breast reconstruction is a safe method with predictable results [17]. In addition, several studies have reported its excellent postoperative outcomes, including reduced pain and animation deformity, because of the preservation of the pectoralis major [11–14].

Several previous studies have discussed prepectoral DTI breast reconstruction. The study by Reitsamer et al. described the full coverage of the prosthesis using porcine ADM Strattice (LifeCell Corporation, Bridgewater, NJ, USA) [4], whereas Vidya et al. reported the beneficial outcomes of a method that fully covers the prosthesis using Braxon^®^, a single large ADM [20]. Similarly, our study reported the outcomes of prepectoral DTI breast reconstruction with complete implant coverage using a double-crossed ADM [9]. In most cases of prepectoral DTI breast reconstruction using ADM, as described in several studies, ADM was used for full coverage of the implant [15, 16].

Some studies have also reported the anterior coverage method, in which only the anterior part of the implant is covered. Kyle et al. introduced a technique for anterior coverage using a method to partially sling AlloDerm™ using the dual-plane technique [20]. Ayesha et al. and Yang et al. reported methods involving the complete anterior coverage of an implant [13, 21], but only a few studies have discussed a method that completely covers the anterior part of the ADM (i.e., complete anterior coverage).

We compared postoperative outcomes between patients who underwent implant insertion through the subpectoral technique and those who underwent implant insertion through the prepectoral technique. No significant difference was noted in major and minor complications between the two groups. However, the mean drain removal period was 10.5429 ± 2.2274 and 8.5588 ± 2.5008 days (p < 0.001) in the control (i.e., subpectoral) and anterior coverage groups, respectively. This implies that recovery after anterior coverage prepectoral implant insertion takes less time compared with the subpectoral technique. The hospitalization period is shortened, and the patient can return to daily life faster because of early discharge. Furthermore, the anterior coverage technique had noninferior stability and faster recovery time than the subpectoral technique.

Moreover, the incidence of skin necrosis is reportedly high while conducting prepectoral reconstruction along with mastectomy skin flap [24]. In our study, the incidence of skin necrosis was lower among those who underwent prepectoral reconstruction using the anterior coverage method than among those who underwent subpectoral reconstruction; however, the difference was not statistically significant. Nevertheless, this is believed to have an impact because surgery was performed only in patients with an indication after confirming that the thickness of the mastectomy flap and the perfusion state were appropriate for prepectoral reconstruction. In addition, because our surgical method covers the anterior surface of the implant after the ADM is spread wide, the relatively good adhesion between the skin flap and ADM may also have an effect.

**Fig. 3:**
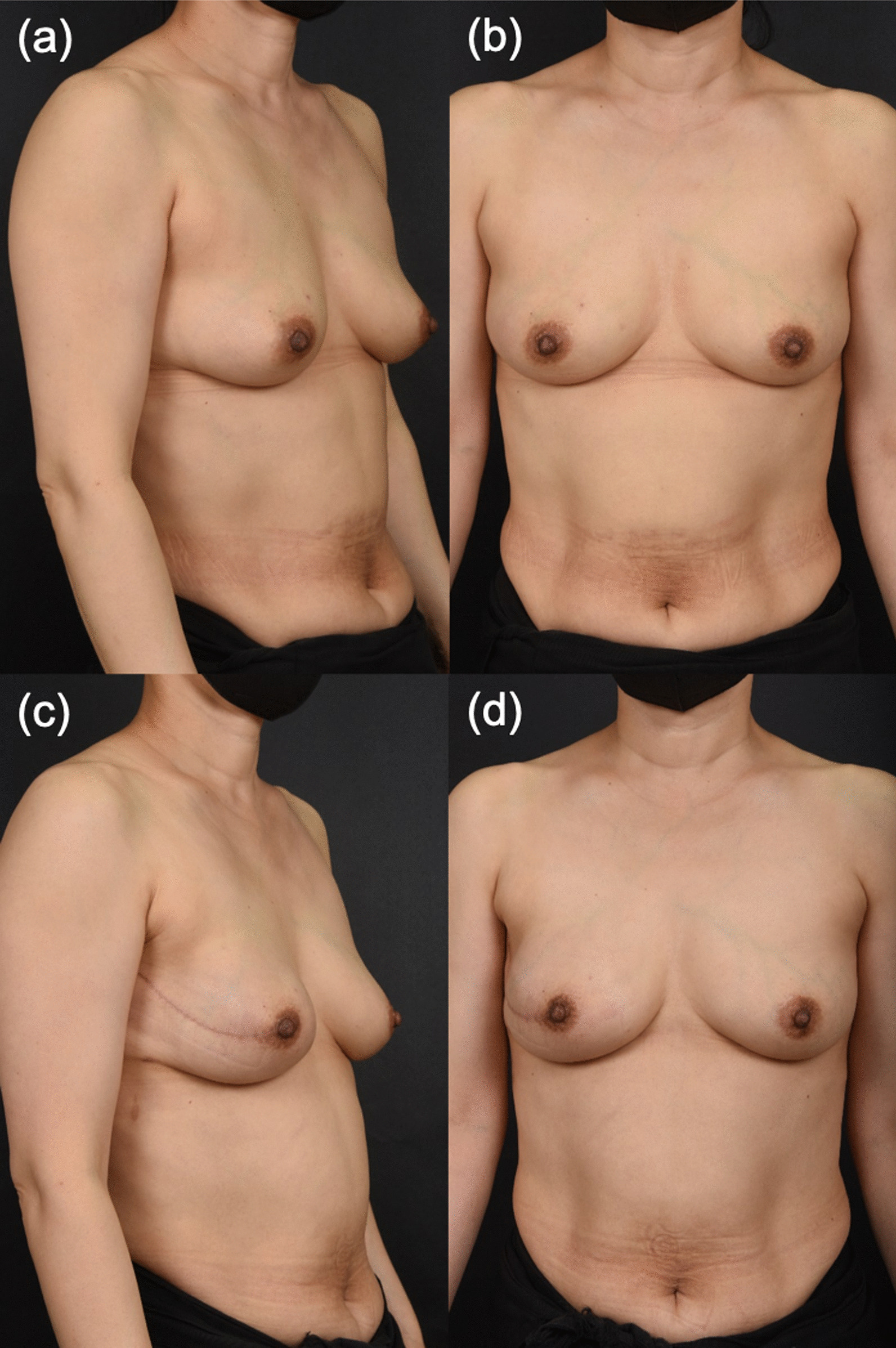
Breast reconstruction with prepectoral implant insertion by anterior coverage (F/49). **a**, **b** Preoperative appearance. **c**, **d** Postoperative appearance at 1 years

One case (2.86%) of capsular contracture occurred in the control (i.e., subpectoral) group. This patient received postmastectomy radiation therapy and underwent subpectoral DTI breast reconstruction. Two patients received postmastectomy radiation therapy in the anterior coverage group, but none of them experienced capsular contracture (Fig. 3).

Infection occurred in only one patient (2.86%) in the control (i.e., subpectoral) group, with no cases of infection (0%) in the anterior coverage group. Despite our compliance with the hospital’s infection prevention protocol while performing breast reconstruction using the DTI technique [13], occasional cases of infection still occur. In such cases, our team immediately administers empirical antibiotics and refers the patient to other departments to rapidly diagnose and establish a treatment plan so that proactive surgical intervention can be considered alongside appropriate drug therapy [22]. In the single case of infection in this study, broad-spectrum antibiotics were administered immediately upon the appearance of symptoms. Salvage reoperation was not performed because the infection symptoms subsided within a short time.

A previous study found that implant visibility and rippling occur frequently after prepectoral DTI breast reconstruction [9]. In this study, rippling occurred slightly more frequently in the anterior coverage group than in the control (i.e., subpectoral) group (two patients [5.71%] vs. zero patients [0%], respectively). As a patient group for prepectoral DTI, an indication is set as a group of patients who had undergone skin-sparing or nipple-sparing mastectomy; flap quality (thickness and vascularity) should be confirmed, and it is advantageous to exclude from the indication for preoperative radiotherapy history, current smokers, and patients with uncontrolled diabetes mellitus in which the skin flap may not be in good condition [9].

No significant difference was noted between the two groups in terms of their subjective satisfaction with breast reconstruction and QoL after surgery, as evaluated using the KNUH Breast-Q scale. Furthermore, no significant differences were observed in breast symmetry measured using the Vectra H2 3D scanner. The Vectra H2 3D scanner enables the measurement of the SN–N distance, IMF–N difference, breast width, N–M distance, breast projection difference, and breast volume of patients (Fig. 2). Yang et al. reported measurements of breast symmetry using surface anatomy [23]. Although there are some discrepancies between their surface anatomy parameters and those used in our study, we did not experience difficulties while measuring breast symmetry using our parameters.

**Fig. 4:**
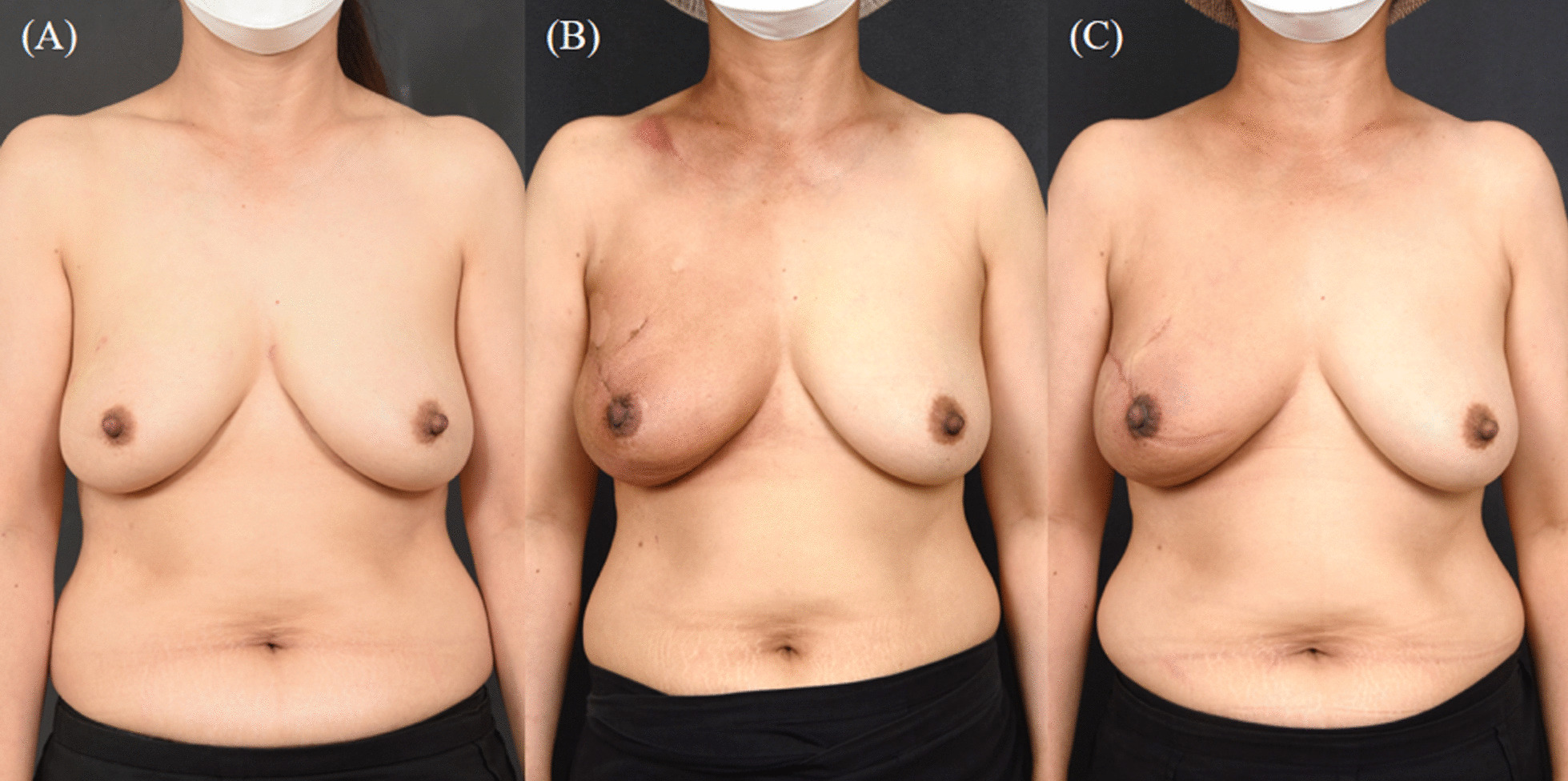
Breast reconstruction with prepectoral implant insertion via anterior coverage (F/51). **a** Preoperative appearance. **b** Postoperative appearance at 5.5 months (immediately after postmastectomy radiotherapy). **c** Postoperative appearance at 12 months (6.5 months after postmastectomy radiotherapy)

According to our results, there was no statistically significant difference in breast symmetry measured using the Vectra H2 3D scanner, but minor differences were observed for all items in the anterior coverage group. This reflects the superiority of anterior coverage in adjusting breast symmetry during breast reconstruction, which is consistent with the findings of existing studies claiming that prepectoral breast reconstruction can yield superior cosmetic outcomes. The superiority of this technique in terms of cosmetic outcomes can be observed in the gross photos (captured during the follow-up) of patients who underwent anterior coverage (Figs. 3, 4).

During prepectoral DTI breast reconstruction with anterior coverage, all aspects of ADM are fixed to the pectoralis muscle. In particular, while fixing its inferior aspect, the ADM is made to form a natural IMF by folding it downward into the implant sizer (Fig. 1); this may improve cases of ptotic breasts. Compared with the prepectoral DTI technique that uses full wrapping, the prepectoral DTI technique with anterior coverage reduces the dead space between the skin flap and ADM. Unlike full wrapping, in which implants are wrapped by ADM based on their shape, the anterior coverage technique takes into consideration the natural breast shape; thus, the ADM is spread over the implant, and the anterior surface of the implant is covered. Complication-related outcomes, such as seroma, were better with the anterior coverage technique than with the full wrapping technique, although these findings require verification in further studies.

Despite its prospective comparative study design, a limitation of this study was the small number of patients in both groups, which hindered statistical verification. Likewise, the difference in implant volume between the two groups is believed to be because of the small number of patients. In addition, the superiority of the anterior coverage method could have been demonstrated in a better manner by comparing it with prepectoral breast reconstruction methods other than the subpectoral method. Finally, the relatively short follow-up period in some patients was considered to be another limitation.

Regardless of these limitations, our findings demonstrated that anterior coverage–based breast reconstruction had safety outcomes compared with the existing subpectoral implant-based reconstruction in terms of faster recovery time and breast symmetry.

## Conclusions

In this study, we reported the safety of the complete anterior coverage implant method using a single, large, square-shaped ADM in DTI breast reconstruction after mastectomy. The safety of this technique is based on its stability, faster recovery time, and cosmetic outcomes compared with subpectoral DTI technique. Prepectoral breast reconstruction with anterior implant coverage using a single, large ADM is a good choice for performing breast reconstruction with implant insertion in patients who have undergone mastectomy.

## Supplementary Information

**Additional file 1: Online Video 1.** The video displays the surgical technique used for prepectoral breast reconstruction with complete anterior implant coverage using a single, large, square-shaped acellular dermal matrix.

## Data Availability

The data that support the findings of this study are available on request from the corresponding author. The data are not publicly available due to privacy or ethical restrictions.

## Abbreviations

DTI: Direct-to-implant
ADM: Acellular dermal matrix
KNUH: Kyungpook National University Hospital
IMF: Inframammary fold
QoL: Quality of life
SN–N: Sternal notch to nipple
IMF–N: Inframammary fold to nipple
N–M: Nipple to midline

## References

[1] American Cancer Society. About breast cancer. Available at: https://www.cancer.org/cancer/breast-cancer/about/howcommon-is-breast-cancer.html. Accessed 12 Jan 2022.

[2] Rhiem K, Schmutzler R (2014). Impact of prophylactic mastectomy in BRCA1/2 mutation carriers. Breast Care.

[3] Albornoz CR, Bach PB, Mehrara BJ (2013). A paradigm shift in U.S. Breast reconstruction: increasing implant rates. Plast Reconstr Surg.

[4] Reitsamer R, Peintinger F (2015). Prepectoral implant placement and complete coverage with porcine acellular dermal matrix: a new technique for direct-to-implant breast reconstruction after nipple-sparing mastectomy. J Plast Reconstr Aesthet Surg.

[5] Ibrahim AM, Koolen PG, Ashraf AA (2015). Acellular dermal matrix in reconstructive breast surgery: survey of current practice among plastic surgeons. Plast Reconstr Surg Glob Open.

[6] Gandhi A, Barr L, Johnson R (2013). Bioprosthetics: changing the landscape for breast reconstruction?. Eur J Surg Oncol.

[7] Macadam SA, Lennox PA (2012). Acellular dermal matrices: use in reconstructive and aesthetic breast surgery. Can J Plast Surg.

[8] Downs RK, Hedges K (2016). An alternative technique for immediate direct-to-implant breast reconstruction—a case series. Plast Reconstr Surg Glob Open.

[9] Lee JS, Kim JS, Lee JH (2019). Prepectoral breast reconstruction with complete implant coverage using double-crossed acellular dermal matrixs. Gland Surg.

[10] Salzberg CA, Ashikari AY, Koch RM, Chabner-Thompson E (2011). An 8-year experience of direct-to-implant immediate breast reconstruction using human acellular dermal matrix (AlloDerm). Plast Reconstr Surg.

[11] Manrique OJ, Banuelos J, Abu-Ghname A (2019). Surgical outcomes of prepectoral versus subpectoral implant-based breast reconstruction in young women. Plast Reconstr Surg Glob Open.

[12] Thangarajah F, Treeter T, Krug B (2019). Comparison of subpectoral versus prepectoral immediate implant reconstruction after skin- and nipple-sparing mastectomy in breast cancer patients: a retrospective hospital-based cohort study. Breast Care (Basel).

[13] Yang JY, Kim CW, Lee JW, Kim SK, Lee SA, Hwang E (2019). Considerations for patient selection: prepectoral versus subpectoral implant-based breast reconstruction. Arch Plast Surg.

[14] Ching AH, Lim K, Sze PW, Ooi A (2022). Quality of life, pain of prepectoral and subpectoral implant-based breast reconstruction with a discussion on cost: a systematic review and meta-analysis. J Plast Reconstr Aesthet Surg.

[15] Vidya R, Iqbal FM (2017). A guide to prepectoral breast reconstruction: a new dimension to implant-based breast reconstruction. Clin Breast Cancer.

[16] Cattelani L, Polotto S, Arcuri MF, Pedrazzi G, Linguadoca C, Bonati E (2018). One-step prepectoral breast reconstruction with dermal matrix-covered implant compared to submuscular implantation: functional and cost evaluation. Clin Breast Cancer.

[17] Sigalove S, Maxwell GP, Sigalove NM (2017). Prepectoral implant-based breast reconstruction: rationale, indications, and preliminary results. Plast Reconstr Surg.

[18] Bernini M, Calabrese C, Cecconi L (2015). Subcutaneous direct-to-implant breast reconstruction: surgical, functional, and aesthetic results after long-term follow-up. Plast Reconstr Surg Glob Open.

[19] Vidya R, Masià J, Cawthorn S (2017). Evaluation of the effectiveness of the prepectoral breast reconstruction with Braxon dermal matrix: First multicenter European report on 100 cases. Breast J.

[20] Chepla KJ, Dagget JR, Soltanian HT (2012). The partial AlloDerm sling: reducing allograft costs associated with breast reconstruction. J Plast Reconstr Aesthet Surg.

[21] Khan A, Tasoulis MK, Teoh V, Tanska A, Edmonds R, Gui G (2021). Pre-pectoral one-stage breast reconstruction with anterior biological acellular dermal matrix coverage. Gland Surg.

[22] Yeo H, Lee D, Kim JS (2021). Strategy for salvaging infected breast implants: lessons from the recovery of seven consecutive patients. Arch Plast Surg.

[23] Yang Y, Mu D, Xu B (2021). An intraoperative measurement method of breast symmetry using three-dimensional scanning technique in reduction mammaplasty. Aesthetic Plast Surg.

[24] Schlenker JD, Bueno RA, Ricketson G, Lynch JB (1978). Loss of silicone implants after subcutaneous mastectomy and reconstruction. Plast Reconstr Surg.

